# Reliability of large language models for advanced head and neck malignancies management: a comparison between ChatGPT 4 and Gemini Advanced

**DOI:** 10.1007/s00405-024-08746-2

**Published:** 2024-05-25

**Authors:** Andrea Lorenzi, Giorgia Pugliese, Antonino Maniaci, Jerome R. Lechien, Fabiana Allevi, Paolo Boscolo-Rizzo, Luigi Angelo Vaira, Alberto Maria Saibene

**Affiliations:** 1 Division of Otolaryngology, Department of Surgical Sciences, Università degli Studi di Torino, Turin, Italy; 2 Otolaryngology Unit, Santi Paolo e Carlo Hospital, Department of Health Sciences, Università degli Studi di Milano, Milan, Italy; 3 Faculty of Medicine and Surgery, “Kore” University of Enna, Enna, Italy; 4 International Federation of Otorhinolaryngological Societies (YO-IFOS) Head and Neck Research Group, Paris, France; 5 Department of Otolaryngology-Head and Neck Surgery, Foch Hospital, School of Medicine, University Paris Saclay, Paris, France; 6 Maxillofacial Surgery Unit, Santi Paolo e Carlo Hospital, Department of Health Sciences, Università degli Studi di Milano, Milan, Italy; 7 Department of Medical, Surgical and Health Sciences, Section of Otolaryngology, University of Trieste, Trieste, Italy; 8 Maxillofacial Surgery Operative Unit, Department of Medicine, Surgery and Pharmacy, University of Sassari, Sassari, Italy; 9 Biomedical Science PhD School, Biomedical Science Department, University of Sassari, Sassari, Italy

**Keywords:** Head and neck cancer, Head and neck oncology, Tongue carcinoma, Laryngeal carcinoma, Oropharyngeal carcinoma, Nasopharyngeal carcinoma, Parotid carcinoma, Oncological diagnosis, Computer-assisted diagnosis, Artificial intelligence, Large language models

## Abstract

**Purpose:**

This study evaluates the efficacy of two advanced Large Language Models (LLMs), OpenAI’s ChatGPT 4 and Google’s Gemini Advanced, in providing treatment recommendations for head and neck oncology cases. The aim is to assess their utility in supporting multidisciplinary oncological evaluations and decision-making processes.

**Methods:**

This comparative analysis examined the responses of ChatGPT 4 and Gemini Advanced to five hypothetical cases of head and neck cancer, each representing a different anatomical subsite. The responses were evaluated against the latest National Comprehensive Cancer Network (NCCN) guidelines by two blinded panels using the total disagreement score (TDS) and the artificial intelligence performance instrument (AIPI). Statistical assessments were performed using the Wilcoxon signed-rank test and the Friedman test.

**Results:**

Both LLMs produced relevant treatment recommendations with ChatGPT 4 generally outperforming Gemini Advanced regarding adherence to guidelines and comprehensive treatment planning. ChatGPT 4 showed higher AIPI scores (median 3 [2–4]) compared to Gemini Advanced (median 2 [2–3]), indicating better overall performance. Notably, inconsistencies were observed in the management of induction chemotherapy and surgical decisions, such as neck dissection.

**Conclusions:**

While both LLMs demonstrated the potential to aid in the multidisciplinary management of head and neck oncology, discrepancies in certain critical areas highlight the need for further refinement. The study supports the growing role of AI in enhancing clinical decision-making but also emphasizes the necessity for continuous updates and validation against current clinical standards to integrate AI into healthcare practices fully.

**Supplementary Information:**

The online version contains supplementary material available at 10.1007/s00405-024-08746-2.

## Introduction

Artificial intelligence is in a continual state of flux. Large language models, or LLMs, represent some of the pioneering technologies designed to change how we interact with information from different domains [1]. OpenAI’s ChatGPT 4 and Google’s Gemini Advanced are two LLMs that demonstrate enormous progress in the area of understanding and generating human-like text and are at the focus of this comparative study, which aims to evaluate these LLMs’ potential as proxies for a multi-domain evaluation in head and neck oncology [2, 3]. Head and neck cancer represents different entities with unique classification and diagnostic criteria. Due to the anatomic diversity, a multi-disciplinary approach to treatment is essential for all patients. Such an approach implies that the experts in various fields work together on a given case. However, the integration of LLMs like ChatGPT 4 and Gemini Advanced gives a new, unexplored avenue of how AI can be used to simplify the integration process, potentially enhancing diagnostic accuracy, treatment planning, and patient outcomes [4–6]. In this context, the recent appearance of a third LLM, Gemini, showcases growing researchers’ interest in how different training routines can impact the quality of LLMs results during medical case analysis. Considering the various factors that may affect the outcomes, it is important to determine whether LLMs are alike or vice versa worse for certain cases, including difficult-to-diagnose oncological cases. The principal aim of the study is to evaluate the accuracy and relevance of the information obtained from LLMs deployment in multidisciplinary oncology cases. By providing them with five different oncology cases from various head and neck subsites for a study and comparing their results with the most recent subsites guidelines, it is possible to understand the LLM’s limitations and opportunities. It is expected that this intervention will help to understand the benefits and drawbacks of LLMs deployment in multidisciplinary oncology cases and improve the patient treatments in this field.

## Methods

### Setting

This study was conceived as a multidisciplinary specialist evaluation of two different LLM treatment suggestions for five different anatomical locations of locoregionally advanced head and neck epithelial malignancies.

This study did not involve human participants, clinical data, or biological material. Therefore, it did not require institutional research ethics committee evaluation.

Two authors (AL and GP) prepared five textual clinical cases. These five Clinical Vignettes were redacted based on real-world cases, though demographics were altered to ensure anonymity. Each case provided an exhaustive clinical narrative, including patient history, surgical pathology diagnosis, clinical evaluation, imaging examination description, and clinical tumor staging. The five cases were designed to provide a general overview of LLM knowledge in different anatomical head and neck subsites (oral cavity, larynx, oropharynx, nasopharynx, salivary glands) presenting advanced epithelial malignancies (four squamous cell carcinoma, and one poorly differentiated carcinoma).

The five cases were submitted on February 29 to ChatGPT 4 (available at https://openai.com/blog/chatgpt from OpenAI, San Francisco, California, US) and Gemini Advanced (available at https://gemini.google.com/ from Alphabet Inc., Mountain View, California, US). Identical prompts (reported in Supplementary Material 1) were used for both chatbots, requesting them to act as the senior head and neck oncologist, chairing an imaginary multidisciplinary oncological group:

“Pretend you are the senior head and neck oncologist, chairing the multidisciplinary oncological group, which is deciding the best treatment for each patient with a new oncological diagnosis. Which treatment would you suggest for the patient thereafter described?”

Each request was entered individually, in a new dialog box and in incognito mode.

### Analysis

The responses generated by each LLM were collected in a Google Documents file (Alphabet Inc., Mountain View, California, US) and sent to two different evaluation panels. Each panel comprised an otolaryngologist and a maxillofacial surgeon. Panel members were informed that the responses they received were AI-generated by two different LLMs, yet they were blinded to the specific LLM responsible for each response. Each panel was assigned a third member to solve disagreements in rating. All raters were instructed to rate the treatment plan proposed according to the latest guidelines issued by the National Comprehensive Cancer Network (NCCN Clinical Practice Guidelines in Oncology for Head and Neck Cancers, version 3.2024), avoiding any personal deviation from the aforementioned guidelines, whether supported or not by newer literature.

The first panel (AMS, FA, and AM for disagreements) was tasked to rate LLM answers via the total disagreement score (TDS) system for LLM medical content [4]. The TDS system rates LLM answers on a scale ranging from 0 (complete agreement) to 12 (complete disagreement) resulting from the partial scores from 0 (no disagreement) to 3 (major disagreement) across four domains: diagnosis, medical management, surgical treatment, and other concerns.

The second panel (PBR, LAV, and JRL for disagreements) was tasked to rate LLM answers via the Quality Assessment of Medical Artificial Intelligence (QAMAI) for LLM medical content [7]. This instrument requires indicating the level of agreement with the LLM answer from 1 (strongly disagree) to 5 (strongly agree) across six domains: accuracy, clarity, relevance, completeness, provision of sources, and usefulness.

Each panel was instructed to agree on a single TDS score for each question and provide a single QAMAI evaluation for each domain and each answer. TDS and QAMAI scores were collected in a Google Sheets file (Alphabet Inc., Mountain View, California, US) and subsequently reassigned to the respective LLM after evaluation for synthesis and statistical analysis.

### Statistics

All statistical tests were performed using SPSS Statistics 28 (IBM Corp., Armonk, New York, US).

TDS and domain related QAMAI scores for each domain and case were considered non-parametric data. Therefore, median and interquartile range (IQR) (reported as median [IQR]) were used as descriptive statistics for continuous data.

TDSs and QAMAI domain-specific scores from ChatGPT 4 and Gemini for each case were compared with a Wilcoxon signed-rank test.

QAMAI domain-specific scores for each case (i.e., for each anatomical subsite) were compared separately via Friedman test for ChatGPT 4 and Gemini answers. Pairwise comparisons between anatomical subsites’ performances were adjusted via Bonferroni correction.* p* values lower than 0.05 were considered statistically significant.

## Results

The responses generated by the two LLMs were reported following the prompt in Online Resource 1 (prompts in bold, ChatGPT 4 replies in regular text, Gemini Advanced replies in italics).

Both LLMs responded pertinently to the prompts, often acknowledging their intrinsic limitations as AIs. In every instance, both ChatGPT and Gemini delivered a thorough analysis of the presented cases, considering not only the primary oncological treatment but also addressing patient support and encouraging the virtual multidisciplinary tumor board to address patients’ desiderata.

The TDSs and domain specific QAMAI scores for each case and for each LLM are reported in Table 1.

**Table 1:** TDS and AIPI domain-specific scores for each LLM and answer

	ChatGPT 4							Gemini Advanced						
	TDS	QAMAI						TDS	QAMAI					
		Acc	Cla	Rel	Com	Ref	Use		Acc	Cla	Rel	Com	Ref	Use
Case 1—oral cavity SCC	3	3	3	4	3	1	3	3	3	4	2	1	1	2
Case 2—larynx SCC	3	3	4	3	2	1	2	4	2	3	2	2	1	2
Case 3—oropharynx SCC	2	4	4	4	4	1	4	0	3	4	3	3	1	3
Case 4—nasopharynx SCC	2	2	3	2	2	1	2	2	3	4	2	2	1	2
Case 5—salivary gland PDC	3	4	5	4	4	1	4	0	3	4	3	2	1	2

*Acc* accuracy, *QAMAI* Quality Assessment of Medical Artificial Intelligence, *Cla* clarity, *Com* completeness, *PCD* poorly differentiated carcinoma, *Rel* relevance, *Ref* reference, *SCC* squamous cell carcinoma, *TDS* total disagreement score, *Use* usefulness

The evaluators’ notes highlighted that the most common critique for both LLMs was the inconsistent use of induction chemotherapy, which was inappropriately suggested for the oral cavity and mentioned as a potential addition for the oro- and nasopharynx. Additionally, the issue of neck dissection was frequently noted. Neither LLM recommended a neck dissection for the surgical treatment of locoregionally advanced laryngeal squamous cell carcinoma (SCC), whereas ChatGPT suggested a selective neck dissection (SND) for a poorly differentiated carcinoma of the salivary glands with confirmed lymph node involvement. Conversely, Gemini was more likely to refrain from proposing a specific treatment after discussing the case (2 out of 5 instances).

### Total disagreement score

The TDS showed a slightly worse agreement towards ChatGPT 4 answers than Gemini (TDS 3 [2, 3] vs. 2 [0–3]), though this difference did not reach statistical significance (Wilcoxon test *p* 0.285). The oropharynx was the subsite consistently associated with the lowest disagreement, while the larynx showed the worst results in terms of TDS. Remarkably, Gemini generated two responses that received a 0 TDS (oropharynx and salivary glands), while at the same time generating the worse reply (larynx). In contrast, ChatGPT's TDS scores were more consistent, typically ranging from 2 to 3 across all subsites.

### Quality assessment of medical artificial intelligence

The AIPI-based comparison between the two LLM performances showed overall better results across all domains for ChatGPT (QAMAI 3 [2–4] vs QAMAI 2 [2, 3] for Gemini, Wilcoxon test *p* 0.004). Using references emerged as the lowest-scoring domain for both LLMs, consistently achieving a score of 1. In contrast, clarity was the highest-scoring domain for both models, with most scores falling between 3 and 4. Notably, the only instance of perfect agreement (5/5) occurred in the salivary glands case, with the response provided by ChatGPT.

When assessing the performances across individual anatomical subsites, ChatGPT showed a higher degree of variability. It achieved high agreement for the oropharynx and salivary glands (respectively QAMAI 4 [1–4] and QAMAI 4 [1–5], while the nasopharynx scored the lowest (QAMAI 2 [1–3]). In contrast, Gemini demonstrated more consistent outcomes, with scores consistently ranging between 2 and 3 across all sites, and the oropharynx being its best-managed case. Upon conducting the Friedman test to compare AIPI scores across different anatomical subsites for each LLM, the results indicated statistically significant differences between ChatGPT (*p* 0.002) and Gemini (*p* 0.028). However, after applying the Bonferroni correction for multiple comparisons, the only significant pairwise difference remained between ChatGPT's performance on the nasopharynx and salivary glands cases.

## Discussion

The evolving role of LLMs in the medical field has generated increasing interest within the scientific community, making the exploration of their potential applications and limitations crucial [1, 8].

Although many studies on the application of AI in the field of otolaryngology are already present in the literature [4, 9, 10], it is only recently that a limited number of articles have begun to emerge, comparing the capabilities of various LLMs in this specific medical domain [4].

To the best of the authors’ knowledge, this study represents the first endeavor in the otolaryngology field to directly compare the capabilities of two advanced LLMs—ChatGPT 4 and Gemini Advanced—specifically within the specialized domain of head and neck oncology. The assessment of clinical case with Gemini is its primary strength because no previous study assessed Gemini in the otolaryngology field. Accordingly, our investigation constitutes a pioneering contribution to the ongoing shift in the scientific paradigm, which positions artificial intelligence as a key player in the future of medicine [11].

This study stands out due to its comparative analysis of the LLMs and as one of the initial attempts to employ externally validated assessment tools specifically crafted for the otolaryngology field [12]. The choice of using two different assessment quality tools to evaluate LLMs’ performances, was based on the subtle difference between the aspects the tools investigate. More specifically, while the TDS score focuses on the clinical approach evaluation, the QAMAI score gives a broader and general assessment of the sanitary information provided, without exploring further clinical aspects (i.e., diagnosis, treatment etc.).

By incorporating such validated instruments, we could identify subtle differences in the responses provided by the models. This method facilitated a detailed examination of the LLMs’ outputs, underlying their potential benefits and limitations in handling complex oncological scenarios. Given the rapidly increasing volume of literature on LLMs, using a standardized assessment tool for evaluating outcomes could streamline the comparison process across different studies, enhancing the consistency and reliability of findings in this burgeoning area of research. For instance, while Gemini showed a slightly better agreement in the TDS, indicating a marginal preference for its answers, ChatGPT outperformed Gemini in the AIPI scores across several domains. This discrepancy underscores the complexity of evaluating AI in healthcare, where different metrics can reveal varied aspects of performance.

The results of our investigation indicate that the LLMs demonstrated commendable performance overall; however, neither achieved optimal outcomes. This shortfall was particularly pronounced in their management of induction chemotherapy—a therapeutic modality that is both controversial and complex. Notably, both LLMs exhibited inconsistencies in the application of this treatment. Specifically, ChatGPT incorrectly recommended induction chemotherapy for cases involving the oral cavity, while Gemini occasionally failed to include specific treatment recommendations. Moreover, the failure to recommend SND in cases of advanced laryngeal SCC highlights a significant deficiency in adherence to established clinical guidelines. This suggests that although these models possess access to extensive data repositories, their capability to apply this information in specialized clinical scenarios precisely remains underdeveloped.

Nevertheless, the models excelled in prioritizing the principles of multidisciplinary team discussions and aligning treatment plans with patient preferences. They skillfully incorporated considerations for patient support, endorsing a holistic approach to treatment planning that mirrors the patient-centric model increasingly advocated in contemporary clinical practice. This adept integration of diverse therapeutic aspects into their recommendations is notable and demonstrates the potential utility of LLMs in facilitating comprehensive care strategies.

Furthermore, the models maintained a robust performance in analyzing patient risk factors, elucidating tumor staging and its implications, thereby providing an objective general assessment of the clinical cases presented. The clinical management and therapeutic strategies suggested were largely pertinent and following the NCCN guidelines. Comparatively, the performance on these oncological topics was superior to that on more specialized subjects—such as odontogenic sinusitis—where the LLM responses revealed a broader spectrum of weaknesses, highlighting several limitations and discrepancies in their outputs [4].

This underscores the critical importance of context, specialization, and the presence of updated and explicit guidelines when evaluating the efficacy of AI in medicine. The observed limitations, such as the consistent error regarding induction chemotherapy and the nuanced oversight concerning specialized procedures like neck dissection, delineate specific areas where LLMs necessitate further refinement. These discrepancies likely arise from the rapidly evolving landscape of medical protocols and the inherent complexities within the field of oncology, where nuanced judgments are frequently essential. The particularity of oncology, characterized by its detailed protocols and swiftly changing guidelines, poses a substantial challenge for LLMs. This highlights the imperative need for continual updates and rigorous validation of these models against the prevailing clinical standards to ensure their relevance and reliability in clinical decision-making.

This preliminary study is subject to certain limitations, primarily associated with the small scale of the analysis, which included only a limited number of cases selected based on uniformity criteria. An examination of a broader range of clinical scenarios and a larger dataset would potentially provide a more comprehensive understanding of the response patterns exhibited by the LLMs. Focusing future research on a single anatomical region within the head and neck may enable the models to handle more cases involving the same oncological pathology, despite variations in clinical presentation and staging. This approach could help elucidate the underlying reasons for the models' weaknesses and illuminate their strengths more effectively.

Another limitation pertains to the evaluation methodologies employed to assess AI responses. The assessment tools used in this research have not been formally validated as a benchmark for comparison. This introduces significant uncertainties in their effectiveness and reliability as instruments for measuring AI performance in medical contexts. As the integration of AI into healthcare progresses, the development and validation of standardized tools that can accurately and reliably assess AI outputs become critical. Moreover, the absence of validated methods means results could vary significantly with different evaluation tools, leading to inconsistencies in how AI technologies are evaluated. This variability could influence the adoption and trust in AI systems within the medical community. Thus, further research is needed to establish standardized, reproducible methods for AI evaluation, ensuring that these technologies can be reliably integrated into clinical practice to improve patient care outcomes.

A further limitation of this study is the methodology employed in the interaction with AI models, specifically regarding submitting prompts and evaluating responses. In our analysis, each prompt was submitted to each AI model, and only the initial response was considered for evaluation. This approach may not adequately capture the variability and adaptability inherent in AI responses. AI systems, especially those underpinned by machine learning technologies, can produce divergent outputs contingent upon minor variations in input or changes in their internal state over time. Evaluating multiple responses to the same prompt could yield a more thorough appraisal of an AI's consistency and reliability. This method would enable researchers to more accurately gauge the spectrum of possible responses an AI might generate and the likelihood of it yielding an optimal or suboptimal outcome.

Furthermore, it would facilitate the identification of patterns of consistency or randomness in AI responses, which are pivotal for establishing trust in AI within clinical decision-making processes. It may also prove advantageous to investigate how variations in the phrasing of identical medical scenarios affect AI performance. In this regard, the lack of information about hyper parameter of Gemini and GPT is an additional limitation because they work as black box. Such inquiry could elucidate the sensitivity of AI models to specific keywords or linguistic structures, a factor of paramount importance in medical settings where precise terminology and detail are essential. Incorporating these methodologies into future studies could markedly enhance our comprehension of AI behavior in clinical contexts, thereby fostering a more robust integration of AI tools in medical practice. These studies would assess the reproducibility of AI responses and aid in developing guidelines for the efficacious utilization of AI in healthcare, ensuring that AI-supported decisions are reliable and clinically pertinent.

## Conclusion

In conclusion, the study highlights to the vital but intricate role of large language models such as ChatGPT 4 and Gemini Advanced in otolaryngology, particularly in head and neck oncology. Despite their robust capability to enable the integration of multidisciplinary care aspects and adhere to the treatment principles of patient-centered care, both models have substantial weaknesses regarding absolute adherence to the clinical guidelines. These weaknesses are evident in the induction chemotherapy and neck dissection cases, and they prove the importance of relentless updating and thorough validation of LLMs to parallel the ever-changing practicability standards. On this note, LLMs appear promising in the improvement of decision-making in healthcare practice, and although their use is warranted, it must be approached with caution because they lack some key competencies, and as earlier suggested, it must be under the guidance of conventional clinical expertise.

## Supplementary Information

Below is the link to the electronic supplementary material.

Supplementary file1 Online Resource 1 - Prompts for Chat GPT and answers generated by the two language models (prompts in bold font, ChatGPT 3.5 answers in plain font, ChatGPT 4 answers in italics). Online Resource 2 - Disagreeement score for each domain, evaluator and answer (DOCX 38 KB)

## Data Availability

All data pertaining to this work are available from the corresponding author upon reasonable request.

## References

[1] Liu S et al (2023) Using AI-generated suggestions from ChatGPT to optimize clinical decision support. J Am Med Inform Assoc 30:1237–1245 37087108 10.1093/jamia/ocad072 PMC10280357

[2] Marchi F, Bellini E, Iandelli A, Sampieri C, Peretti G (2024) Exploring the landscape of AI-assisted decision-making in head and neck cancer treatment: a comparative analysis of NCCN guidelines and ChatGPT responses. Eur Arch Otorhinolaryngol 281:2123–2136 38421392 10.1007/s00405-024-08525-z

[3] Sarma G, Kashyap H, Medhi PP (2024) ChatGPT in head and neck oncology-opportunities and challenges. Indian J Otolaryngol Head Neck Surg 76:1425–1429 38440617 10.1007/s12070-023-04201-6 PMC10908741

[4] Saibene AM et al (2024) Reliability of large language models in managing odontogenic sinusitis clinical scenarios: a preliminary multidisciplinary evaluation. Eur Arch Otorhinolaryngol 281:1835–1841 38189967 10.1007/s00405-023-08372-4 PMC10943141

[5] Vaira LA et al (2023) Accuracy of ChatGPT-generated information on head and neck and oromaxillofacial surgery: a multicenter collaborative analysis. Otolaryngol Head Neck Surg. 10.1002/ohn.489 37595113 10.1002/ohn.489

[6] Lechien JR et al (2024) Performance and consistency of ChatGPT-4 versus otolaryngologists: a clinical case series. Otolaryngol Head Neck Surg. 10.1002/ohn.759 38591726 10.1002/ohn.759

[7] Vaira LA et al. QAMAI. Eur. Arch. Otorhinolaryngol. (being published) 10.1007/s00405-024-08710-0

[8] Liao Z, Wang J, Shi Z, Lu L, Tabata H (2024) Revolutionary potential of ChatGPT in constructing intelligent clinical decision support systems. Ann Biomed Eng 52:125–129 37332008 10.1007/s10439-023-03288-w

[9] Mäkitie AA et al (2023) Artificial intelligence in head and neck cancer: a systematic review of systematic reviews. Adv Ther 40:3360–3380 37291378 10.1007/s12325-023-02527-9 PMC10329964

[10] Bulfamante AM et al (2023) Artificial intelligence, machine learning, and deep learning in rhinology: a systematic review. Eur Arch Otorhinolaryngol 280:529–542 36260141 10.1007/s00405-022-07701-3 PMC9849161

[11] Lechien JR (2024) Generative artificial intelligence in otolaryngology-head and neck surgery editorial: be an actor of the future or follower. Eur Arch Otorhinolaryngol 281:2051–2053 38407611 10.1007/s00405-024-08579-z

[12] Lechien JR et al (2024) Validity and reliability of an instrument evaluating the performance of intelligent chatbot: the Artificial Intelligence Performance Instrument (AIPI). Eur Arch Otorhinolaryngol 281:2063–2079 37698703 10.1007/s00405-023-08219-y

