# Supplementary material for: Reliability of large language models for advanced head and neck malignancies management: a comparison between ChatGPT 4 and Gemini Advanced
Source: Eur Arch Otorhinolaryngol. 2024 May 25;281(9):5001–6. doi: 10.1007/s00405-024-08746-2 (PMC11392976; doi:10.1007/s00405-024-08746-2)
Supplement: Supplementary file 1 — Supplementary file1 Online Resource 1 - Prompts for Chat GPT and answers generated by the two language models (prompts in bold font, ChatGPT 3.5 answers in plain font, ChatGPT 4 answers in italics). Online Resource 2 - Disagreeement score for each domain, evaluator and answer (DOCX 38 KB) [file 405_2024_8746_MOESM1_ESM.docx]

**CASE 1: ORAL CAVITY**

**Pretend you are the senior head and neck oncologist, chairing the multidisciplinary oncological group, which is deciding the best treatment for each patient with a new oncological diagnosis. Which treatment would you suggest for the patient thereafter described?**

**The patient is a 57-year-old male recently diagnosed with squamous cell carcinoma of the left half of the tongue. Tumor staging is cT4aN2bM0. The patient is an active smoker (20 pack years) and has a daily alcohol intake of 3 standard units. The patient has no other significant active disease and his medical record reports no previous surgeries, treatments, or significant conditions.**

**The physical examination shows, on the left side of the tongue, a lesion mildly ulcerated, with irregular margins, without any active bleeding. The imaging performed for the tumor is as follows. Contrast-enhanced magnetic resonance imaging report is the following (in quotation marks) “A focal alteration with an ulcerated component is observed at the left lingual margin, with an overall maximum diameter of approximately 5 cm (anteroposterior diameter) and an estimated depth of invasion (DOI) of about 1.6 cm. The alteration affects the extrinsic lingual musculature of the involved side, infiltrating the genioglossus muscle, the hyoglossus muscle, and the caudal portion of the styloglossus and palatoglossus muscles. The sublingual space is partially involved. No evidence of bone erosions. Presence of several enlarged lymph nodes at the right level II, the largest with a diameter of about 1 cm. No evidence of extracapsular extension.” Contrast-enhanced computed tomography report is the following (in quotation marks) “NECK: At the level of the left half of the tongue, there is an area of increased contrast uptake (dimensions approximately AP x LL x CC: 45 x 18 x 45 mm) that is the site of a known heteroplastic lesion, ulcerated, with involvement of the ipsilateral hypoglossal and genioglossal muscles, abutting and without a clear cleavage plane with the ipsilateral mylohyoid and geniohyoid muscles, without encroachment into the contralateral half of the tongue nor evidence of bone involvement. Several heterogeneous oval lymphadenopathies, likely secondary, are detected in the left lateral-cervical area, the largest at level II (21x14 mm) and level III (20x10 mm). No densitometric alterations in the parotid or submandibular glands. Preserved patency of the parapharyngeal fat spaces. Micro-calcification at the lower pole of the left thyroid lobe. CHEST: No pulmonary lesions suspicious for metastasis are evident. Some areas of faint ground-glass consolidation in the apical-dorsal portions of the upper lobes, primarily hypothesized as minor inflammatory phenomena, are to be re-evaluated over time. No pleural effusion nor significant pericardial effusion. Several lymphadenopathies with a short axis slightly over 1 cm are detected in the right hilar region (max 14x13 mm) and others with a short axis under 1 cm in the mediastinum (the largest in the right paratracheal area of 13 x 8 mm and in the Barety's lodge of 14 x 9 mm). BONE: No evident focal bony alterations suspicious for secondary involvement in the bone segments included in the cervical-thoracic scan planes.”**

*This patient's case presents a complex clinical scenario with a locally advanced head and neck cancer requiring careful and strategic treatment planning. Here's a breakdown of factors to consider:*

- *Tumor Staging: cT4aN2bM0 indicates a large primary tumor (T4a) with substantial lymph node involvement (N2b) but no evidence of distant metastasis (M0).*
- *Risk Factors: The patient's smoking and alcohol use increase their risk of recurrence and overall treatment challenges.*
- *General Health: No significant comorbidities are mentioned, potentially improving the patient's tolerance for aggressive treatment.*
- *Imaging: Comprehensive imaging provides detailed tumor mapping and nodal involvement.*

*Multidisciplinary Approach is Critical*

*The complexity of this case requires input from:*

- *Head and Neck Surgeon: To assess the feasibility and extent of surgical resection, as well as risks involved.*
- *Radiation Oncologist: To determine the role of radiation therapy, both as primary treatment or in the post-operative setting, and discuss potential side effects.*
- *Medical Oncologist: To evaluate if chemotherapy will be beneficial (before surgery, concurrently with radiation, or after).*

*Treatment Recommendations*

*Based on the information provided, here's a likely treatment approach:*

1. *Initial Discussion: The multidisciplinary team will meet to discuss recommendations, potential side effects, and the patient's preferences. Smoking and alcohol cessation would be strongly advised.*
2. *Combination Therapy is Likely: Given the tumor stage, a combination of treatment modalities is probable:*
   - *Surgery: Surgical resection is likely the primary treatment, seeking to remove the tumor and involved lymph nodes. The extent of surgery depends on surgical assessment.*
   - *Radiation Therapy: Used either before surgery (neoadjuvant) to shrink the tumor or after surgery (adjuvant) to reduce the risk of recurrence.*
   - *Chemotherapy: May be added concurrently with radiation (chemoradiation) to improve treatment response and decrease recurrence risk.*

*Specifics are Case-Dependent*

*The exact treatment plan will depend on further discussions within the multidisciplinary team and the patient's preferences. Factors like:*

- *Surgical Assessment: Specific surgical techniques and the potential need for reconstructive surgery.*
- *Radiation Planning: Detailed radiation treatment plans, dosages, and focus areas.*
- *Chemotherapy Choice: Specific chemotherapy agents and their potential side effects.*

*The patient will play a large role in shared decision-making. Their values, concerns about potential side effects, and overall quality of life must be weighed alongside the best clinical approach to the advanced cancer.*

*Disclaimer: I'm an AI language model, not a doctor. This information is not a substitute for professional medical advice. It's essential for the patient to have in-depth consultations with their treating physicians.*

Given the detailed description of the patient's condition and the imaging reports, the patient has an advanced stage squamous cell carcinoma of the tongue (cT4aN2bM0). This stage indicates a large primary tumor (T4a) with significant local invasion, involvement of multiple ipsilateral lymph nodes (N2b), but no distant metastasis (M0). The patient's history of smoking and alcohol use are significant risk factors for head and neck cancers and can influence the prognosis and treatment response.

Recommended Treatment Approach:

1. Multimodal Therapy: For a patient with this stage of oral squamous cell carcinoma, a combination of treatments is often most effective. Given the size of the tumor, its depth of invasion, and lymph node involvement, a multimodal approach including surgery, radiation therapy, and chemotherapy should be considered.
2. Surgery: The primary treatment for this stage might involve a wide excision of the tumor, including partial glossectomy (removal of part of the tongue) to achieve clear margins. Given the involvement of extrinsic muscles of the tongue and the lymph node metastasis, a neck dissection to remove lymph nodes in the affected areas would also be indicated.
3. Radiation Therapy: Postoperative radiation therapy is recommended to address microscopic disease and improve local control, especially given the tumor's extensive local invasion and lymph node involvement. Intensity-modulated radiation therapy (IMRT) could be considered to minimize damage to surrounding healthy tissues.
4. Chemotherapy: Concurrent chemoradiation, possibly with agents like cisplatin, may be recommended as part of the treatment plan to enhance the effectiveness of radiation therapy and to address the risk of distant metastasis. Chemotherapy could also be considered as neoadjuvant (pre-surgery) to reduce the tumor size, potentially improving surgical outcomes.
5. Targeted Therapy and Immunotherapy: Depending on the molecular characteristics of the tumor, targeted therapies or immunotherapy might be options. For example, if the tumor expresses PD-L1, immunotherapy agents like pembrolizumab could be considered.
6. Supportive Care: Given the potential impact on speech, swallowing, and nutrition, a multidisciplinary approach including speech and language therapy, nutritional support, and possibly psychological support is crucial for optimal rehabilitation.
7. Follow-up and Monitoring: Regular follow-up is necessary to monitor for recurrence, manage side effects, and address any functional impairments resulting from treatment.
8. Lifestyle Modifications: Advising the patient to quit smoking and reduce alcohol intake is crucial, as these are significant risk factors for recurrence and secondary primary tumors.

This treatment plan should be discussed in detail with the patient, considering his overall health, preferences, and the potential impact on his quality of life. The multidisciplinary team approach, including oncologists, surgeons, radiologists, pathologists, nursing staff, and rehabilitation specialists, is essential for comprehensive care.

**CASE 2: LARYNX**

**Pretend you are the senior head and neck oncologist, chairing the multidisciplinary oncological group, which is deciding the best treatment for each patient with a new oncological diagnosis. Which treatment would you suggest for the patient thereafter described?**

**The patient is a 66-year-old woman with a recent diagnosis of squamous cell carcinoma of the larynx (right vocal cord and right laryngeal ventricle). Tumor staging is cT4a cN2b cM0. The patient is an active smoker and has no history of alcohol consumption. She has had a recent left cerebral ischemic stroke with sequelae of right motor hemisyndrome, aphasia, and dysphagia. Her medical record reports mixed anxiety depressive disorder and anorexia. The endoscopy shows a lesion, suggestive of heteroplasmy, of about 10 mm originating on the right vocal cord, occupying the ipsilateral laryngeal ventricle and extending posteriorly towards the median line. The motility of the vocal cords is maintained. The imaging performed for the tumor is as follows. Contrast-enhanced computed tomography report is the following (in quotation marks): “Presence of an expansive, evolutionary-looking lesion on the right vocal cord, which also occupies the homolateral laryngeal ventricle and posteriorly exceeds the median line. The lesion is poorly delineated but has a thickness measurable at approximately 10 mm. On the same side of the lesion the thyroid cartilage appears discontinuous and contrast enhancement can be seen outside the cartilage, displacing the laryngeal strap muscles. The piriform sinuses are patent. Two enlarged lymph nodes on the right third level of the neck appear pathological, while other lymph nodes with an oval morphology appear reactive, with a short axis of less than a centimeter, at various levels of the lateral cervical areas on both sides. The parapharyngeal fat spaces are patent and symmetrical. The parotid glands appear regular. There is an asymmetric representation of the submandibular salivary glands due to the smaller size on the left. Centrilobular emphysema is more evident in the upper lobes, with fibro-scarring outcomes at the apical segments. Some segmental and subsegmental bronchial branches in the lower lobes have slightly thickened walls on a probable basis of chronic inflammation and are obliterated by material likely mucous. Some bilateral subsolid pulmonary nodules, the largest subpleural at the anterior segment of the right lower lobe (5 mm), are nonspecific and worthy of reevaluation. No pleural effusion. Modest pericardial effusion (maximum thickness of about 11 mm). No mediastinal and axillary lymphadenopathy.”**

*This case presents a highly complex scenario due to the patient's advanced laryngeal cancer, her recent stroke with resulting disabilities, and her underlying mental health conditions. Here's how I would approach treatment recommendations as the senior head and neck oncologist:*

*Critical Considerations*

- *Tumor Staging: cT4aN2bM0 indicates a large and invasive laryngeal tumor (T4a) with substantial lymph node involvement (N2b) but no distant metastasis (M0).*
- *Stroke Sequelae: The patient's right-sided weakness (hemiparesis), speech difficulties (aphasia), and swallowing issues (dysphagia) severely impact treatment options and potential side effects.*
- *Mental Health: Existing anxiety, depression, and anorexia require careful attention in the patient's care, as they can worsen under cancer treatment stress.*
- *Smoking: Continued smoking poses risks to treatment efficacy and wound healing.*

*Multidisciplinary Team is Essential*

*This case demands close collaboration between:*

- *Head and Neck Surgeon: Assess the feasibility of surgery and the potential need for a total laryngectomy (removal of the voice box), along with reconstructive options.*
- *Radiation Oncologist: Determine if radiation therapy is possible with the patient's current condition, address any potential complications due to the stroke, and discuss side effects.*
- *Medical Oncologist: Evaluate potential chemotherapy options, given the patient's overall health and ability to tolerate treatment.*
- *Speech and Language Pathologist: Assess the patient's current swallowing and communication abilities, predict rehabilitation needs, and suggest alternative communication methods if necessary.*
- *Nutritionist: Evaluate the patient's nutritional status, devise a plan to address anorexia, and support feeding needs, possibly requiring a feeding tube.*
- *Psychiatrist/Psychologist: Manage the patient's mental health, address anxiety and depression, and provide support during treatment.*

*Treatment Considerations*

*Here's a likely approach, balancing oncologic goals with the patient's overall condition:*

1. *Patient and Family Discussion: Candid conversations about treatment goals, realistic expectations, and possible outcomes are crucial given the patient's state.*
2. *Supportive Care: Addressing immediate needs such as communication, nutrition, and mental health takes priority. Speech therapy and nutritional support might be needed before starting any major treatment.*
3. *Smoking Cessation: This is critical for treatment success and overall health, requiring strong support.*
4. *Treatment Options:*
   - *Limited Surgery: Resection of the tumor may be considered if deemed feasible but will depend on the patient's general condition and potential for functional recovery.*
   - *Chemoradiation: While a standard approach for advanced laryngeal cancer, it poses significant risks with the patient's swallowing issues and could lead to severe aspiration.*
   - *Palliative Care: Since the goal of cure might be unrealistic, palliative treatment to control symptoms and improve quality of life may be the most appropriate path.*

*Decision-Making*

*Arriving at the best treatment plan will be a process, not a single decision. It requires ongoing assessment, close communication with the patient and their family, and adjusting plans as needed.*

*The patient's quality of life and well-being must be at the forefront of all decisions made alongside their family's input.*

*Disclaimer: I'm an AI language model, not a doctor. This information is not a substitute for professional medical advice. Consultations with the patient's treating physicians are essential.*

Given the complexity of the case you've described, the treatment plan for this patient requires careful consideration of several factors, including the tumor's stage, the patient's overall health, and the potential impact of treatment options on her quality of life. The patient has a squamous cell carcinoma of the larynx with a staging of cT4a cN2b cM0, indicating a locally advanced tumor with regional lymph node involvement but no distant metastasis. Her medical history, including a recent stroke with significant sequelae and a mixed anxiety-depressive disorder, complicates the treatment approach.

Multidisciplinary Team Considerations

1. Tumor Board Review: The case should be discussed in a multidisciplinary tumor board that includes head and neck surgeons, medical oncologists, radiation oncologists, radiologists, pathologists, rehabilitation specialists, and speech and swallowing therapists. This team can provide a comprehensive evaluation of the patient's condition and collaboratively decide on the best treatment approach.
2. Treatment Options:
   - Surgery: Given the T4a classification, surgery could be considered; however, the patient's recent stroke and its sequelae might limit her ability to tolerate a major surgical procedure. A partial or total laryngectomy could be necessary, but the impact on her quality of life, particularly her ability to speak and swallow, must be carefully considered.
   - Radiation Therapy: Intensity-modulated radiation therapy (IMRT) or volumetric modulated arc therapy (VMAT) could be options to target the tumor while sparing surrounding healthy tissue. Given her dysphagia and risk of further swallowing difficulties, careful planning is required to minimize exposure to critical structures.
   - Chemotherapy: Concurrent chemoradiation with agents like cisplatin may enhance the efficacy of radiation therapy but also increases the risk of toxicities. The patient's overall health and ability to tolerate chemotherapy must be assessed.
   - Targeted Therapy and Immunotherapy: For patients with specific genetic markers or in cases where conventional treatments are contraindicated, targeted therapies or immunotherapy might be options. Biomarker testing of the tumor could provide insights into potential targeted treatments.
3. Supportive Care: Given her history of anorexia and mixed anxiety-depressive disorder, supportive care including nutritional support, psychological counseling, and rehabilitation services will be crucial regardless of the chosen treatment path.
4. Smoking Cessation: Advising the patient to quit smoking is essential, as continued smoking can affect treatment outcomes and overall prognosis.

Decision-Making Process

- Patient Preferences: It's important to discuss the potential benefits and side effects of each treatment option with the patient, considering her current quality of life and treatment preferences.
- Functional Preservation: Given the patient's existing conditions, treatments that preserve laryngeal function and minimize impact on swallowing and speech should be prioritized, if clinically feasible.
- Comorbidities: The patient's stroke history and its neurological sequelae, along with her psychological condition, must be factored into the decision-making process, aiming for a treatment plan that is both effective and tolerable.

Conclusion

In summary, a tailored approach that balances oncological control with the preservation of quality of life is essential. The multidisciplinary team's recommendation will likely lean towards a combination of radiation therapy and chemotherapy, considering the patient's complex medical history and the need to preserve laryngeal function. However, the final decision should be made in close consultation with the patient, fully considering her values, preferences, and the potential impact on her quality of life.

**CASE 3: OROPHARYNX**

**Pretend you are the senior head and neck oncologist, chairing the multidisciplinary oncological group, which is deciding the best treatment for each patient with a new oncological diagnosis. Which treatment would you suggest for the patient thereafter described? The patient is a 55 years old man, with a recent diagnosis of squamous cell carcinoma of the oropharynx. The immunohistochemical analysis of the sample is negative for p16. Tumor staging is cT1 cN2c cM0. The patient has no history of smoking or alcohol consumption. He has no other significant active disease and his medical record reports no previous surgeries, treatments, or significant conditions. The physical examination shows right tonsillar swelling extending superiorly towards the soft palate; on palpation, a 5 cm swelling of hard consistency, fixed in relation to both superficial and deep planes is present on the right side at the I laterocervical level. The imaging performed for the tumor is as follows. Contrast-enhanced computed tomography report is the following (in quotation marks): “In the oral cavity, posteriorly and on the right, at the transition between the oro- and hypopharyngeal region on the right, there is a heterogeneous asymmetric alteration measuring 15 mm with enhancement. Suspicious lymphadenopathy with a short axis of up to one centimeter is associated with the lateral cervical and submandibular regions, especially on the right. No suspicious focal lesions suggestive of involvement of the major salivary glands are evident. The chest is negative for metastatic lesions.”**

*Here's a breakdown of the case and a multidisciplinary approach to treatment recommendations, taking your role as senior head and neck oncologist into account:*

*Case Summary*

- *Patient: 55-year-old male*
- *Diagnosis: Squamous cell carcinoma of the oropharynx, p16 negative*
- *Staging: cT1 cN2c cM0 (Early primary tumor, significant regional lymph node involvement, no distant metastasis)*
- *Other considerations: Non-smoker, no significant medical history*

*Multidisciplinary Team (MDT) Considerations*

*As the chair of the MDT, you'll work with specialists across disciplines including:*

- *Surgery: Evaluate the feasibility of surgical resection with curative intent.*
- *Radiation Oncology: Assess suitability for various radiation therapy options, including definitive radiotherapy, adjuvant radiotherapy, or a combination depending on surgical outcomes.*
- *Medical Oncology: Consider systemic therapies like chemotherapy and/or immunotherapy, potentially in a neoadjuvant (before surgery) or adjuvant (after surgery/radiation) setting.*
- *Other Specialists:*
  - *Speech and language therapy for pre-and post-treatment support.*
  - *Dietician to address potential nutritional issues during and after treatment.*
  - *Dentistry for pre-treatment dental evaluation and treatment optimization.*

*Treatment Recommendations*

*Since this is a p16-negative oropharyngeal cancer, the likelihood of it being associated with the HPV virus is low. This influences treatment decisions:*

- *Surgery followed by adjuvant radiation therapy (with or without chemotherapy): This is often a preferred approach, especially since the primary tumor is relatively small (cT1). The extent of surgery (wide local excision, transoral robotic surgery, etc.) would depend on the tumor's location and accessibility. Neck dissection would be necessary to address the lymph node involvement.*
- *Definitive (curative) chemoradiation: May be a good option if surgery is not feasible or has a high risk of complications. It involves combining chemotherapy with a full course of radiation therapy.*
- *Clinical trials: The patient might be eligible for clinical trials investigating newer treatment approaches or immunotherapy combinations, especially given the p16-negative status.*

*Important Considerations:*

- *Patient preference: Thoroughly discuss the different treatment options, their risks, benefits, and impact on quality of life to help the patient make an informed decision.*
- *Performance status: Evaluate the patient's overall health and fitness to tolerate potentially aggressive treatments.*

*Disclaimer*

*I am an AI language model and cannot provide definitive medical advice. This response is for informational purposes only. The best treatment plan for this patient should be determined through a comprehensive consultation with their multidisciplinary cancer care team.*

*Let me know if you'd like a more detailed breakdown of any of the specific treatment options!*

Given the detailed case description of a 55-year-old man with a recent diagnosis of squamous cell carcinoma of the oropharynx, negative for p16, and staged as cT1 cN2c cM0, the following treatment approach would be considered based on current guidelines and evidence-based practices in head and neck oncology:

1. Treatment Overview

For a patient with p16-negative oropharyngeal squamous cell carcinoma (OPSCC), the prognosis and treatment approach differ significantly from p16-positive cases. P16 negativity often indicates a non-HPV-related cancer, which is typically associated with a poorer prognosis and may respond differently to treatment. Given the staging of cT1 cN2c cM0, the cancer is localized to the oropharynx with significant lymph node involvement but no distant metastasis.

2. Primary Treatment Options

a. Surgery:

- Transoral robotic surgery (TORS) or transoral laser microsurgery (TLM): Given the tumor's location and size, if technically feasible, TORS or TLM could be considered to remove the primary tumor with minimal morbidity. However, the extent of lymph node involvement (N2c) necessitates a comprehensive neck dissection, likely on both sides due to bilateral nodal disease.

b. Radiation Therapy:

- Primary Radiotherapy (RT) with or without chemotherapy: For patients who are not surgical candidates or prefer non-surgical management, intensity-modulated radiation therapy (IMRT) is a standard approach. Given the N2c neck, concurrent chemoradiation with a platinum-based agent (e.g., cisplatin) is recommended to enhance the treatment's efficacy, considering the patient's good performance status and absence of significant comorbidities.

3. Adjuvant Treatment:

- If surgery is chosen and depending on the final pathology report (presence of extracapsular spread, positive margins), adjuvant radiation therapy with or without chemotherapy may be indicated to reduce the risk of locoregional recurrence.

4. Consideration of Clinical Trials:

- Enrollment in clinical trials should be considered if available, offering the patient access to novel therapies or treatment combinations that could potentially offer better outcomes or reduced side effects.

5. Supportive Care:

- Regardless of the treatment modality, supportive care including nutritional support, speech and swallowing therapy, and management of treatment-related side effects is crucial for maintaining quality of life.

6. Follow-Up and Surveillance:

- Regular follow-up with imaging and physical exams to monitor for recurrence or metastasis, as well as secondary primary tumors, given the patient's history of squamous cell carcinoma.

Decision-Making Considerations:

- The choice between surgery and chemoradiation should be made after discussing the potential benefits, risks, and side effects of each treatment option with the patient, considering his preferences, quality of life considerations, and the multidisciplinary team's input.

This treatment plan is based on the current understanding and guidelines in head and neck oncology. It's important to note that treatment decisions should be individualized, taking into account the latest research, clinical trials, and the patient's overall health, preferences, and social support system.

**CASE 4: NASOPHARYNX**

**Pretend you are the senior head and neck oncologist, chairing the multidisciplinary oncological group, which is deciding the best treatment for each patient with a new oncological diagnosis. Which treatment would you suggest for the patient thereafter described? The patient is a 45-year-old man with a recent diagnosis of squamous cell carcinoma of the nasopharynx. Tumor staging is cT1 cN2c cM0. The patient has no history of smoking or alcohol consumption. The patient has no other significant active disease and his medical record reports no previous surgeries, treatments, or significant conditions. The imaging performed for the tumor is as follows.**

**Contrast-enhanced computed tomography report is the following (in quotation marks): “A lesion is present occupying the right nasopharyngeal vault and the ipsilateral parapharyngeal tissues, protruding into the ipsilateral sphenoid sinus, involving the cavernous sinus, the apex of the petrous bone with the cochlea and labyrinth preserved, and the clivus with an erosion of the cortical bone and adjacent trabecular irregularities with trabecular thinning interspersed with pseudosclerosis.” Contrast-enhanced magnetic resonance imaging report is the following (in quotation marks): “Presence of an expansive lesion infiltrating the mucosa of the right pharyngeal vault, preliminarily compatible with nasopharyngeal carcinoma. Specifically, there is involvement of the salpingopharyngeal fold, Rosenmuller's fossa, torus tubarius, and the Eustachian tube. On the right side, the carotid canal, Vidian canal, cavernous sinus, and Meckel's cave are affected; the latter two structures show thickening of their content post-contrast as indicative of perineural spread of the disease.”**

*Here's a breakdown of the situation and potential treatment recommendations for the patient, considering the context of a multidisciplinary oncology group:*

*Understanding the Case*

- *Patient Profile: A 45-year-old male with no significant prior medical history. This is somewhat unusual for nasopharyngeal cancer, as it's more often associated with factors like Epstein-Barr Virus (EBV) infection, smoking, and alcohol use.*
- *Cancer Details: Squamous cell carcinoma of the nasopharynx, which is a type of head and neck cancer.*
- *Staging: cT1 cN2c cM0*
  - *cT1: Tumor is relatively small and localized*
  - *cN2c: Cancer has spread to lymph nodes on both sides of the neck*
  - *cM0: No distant metastasis*
- *Imaging: Shows extensive local spread. The tumor has invaded nearby structures, including the cavernous sinus (critical for nerves and blood vessels) and is eroding bone.*

*Treatment Considerations*

*The standard of care for this stage of nasopharyngeal carcinoma typically involves a combination of:*

- *Concurrent Chemoradiation Therapy (CCRT): Radiation therapy delivered simultaneously with chemotherapy. This is the primary treatment modality to destroy the tumor and address the spread to lymph nodes.*
- *Induction Chemotherapy: Chemotherapy given before CCRT might be considered to further shrink the tumor, especially given its size and complex involvement of surrounding structures.*
- *Surgery: Surgery is usually not the first option with this stage, but may be considered in certain cases:*
  - *Neck dissection: To remove affected lymph nodes if they persist after CCRT.*
  - *Limited resection: If residual tumor remains after treatment and can be safely removed.*

*Multidisciplinary Team's Role*

*The decision is complex and requires expertise from various specialists:*

- *Radiation oncologist: To design the radiation treatment plan, dosage, technique (IMRT or proton beam are likely needed), and manage associated side effects.*
- *Medical oncologist: To determine the chemotherapy regimen (cisplatin-based is common), timing, and management of potential side effects.*
- *Head and neck surgeon: To assess if surgery has a role pre- or post-treatment.*
- *Other specialists: Dentist, dietician, speech pathologist should be involved for pretreatment preparations and support during/after treatment.*

*Discussion Points for Your Role*

*As the chair, you'd need to:*

1. *Present the case summary: Highlighting relevant details and imaging findings.*
2. *Guide discussion: Seek input from each specialist on their recommended approach and any specific considerations.*
3. *Facilitate consensus: Explore potential treatment sequences (e.g., induction chemo followed by CCRT vs. CCRT alone) and raise any concerns about potential toxicities and management.*
4. *Address patient-specific factors: Consider the patient's preferences, potential side effects, and support network.*

*Disclaimer: This response is for informational purposes only and does not constitute medical advice. Real-world treatment decisions are highly individualized and involve extensive discussions between doctors and patients.*

The information provided indicates that the patient has a stage III nasopharyngeal carcinoma (NPC), given the cT1 cN2c cM0 staging. This means the tumor is localized to the nasopharynx (T1), there are bilateral metastatic lymph nodes, none greater than 6 cm in size (N2c), and there's no distant metastasis (M0).

For a non-metastatic, stage III NPC patient who is otherwise healthy, the standard treatment approach would typically include:

1. Radiotherapy: Intensity-Modulated Radiation Therapy (IMRT) is the preferred method as it allows for focused radiation beams to the tumor and involved nodes, minimizing damage to surrounding tissues.
2. Chemotherapy: Concurrent chemoradiotherapy with a platinum-based agent (such as cisplatin) is recommended as it has been shown to improve survival rates in comparison to radiotherapy alone. The chemotherapy acts as a radiosensitizer, making the cancer cells more vulnerable to radiation.

Given the involvement of critical structures such as the cavernous sinus, carotid canal, and potential perineural spread, aggressive local treatment is necessary to achieve control. Since there's no distant metastasis, systemic treatment would aim to address regional control and prevent distant spread.

The multidisciplinary team (MDT) would also include specialists in radiology, medical oncology, radiation oncology, head and neck surgery, pathology, and potentially a neurosurgeon given the proximity to the skull base. Other considerations may include:

- Targeted therapy: Depending on the expression of specific biomarkers, targeted therapies could be considered.
- Immunotherapy: Emerging evidence suggests a role for immunotherapy in the treatment of head and neck cancers, including NPC.
- Nutrition and Speech Therapy: Given the location of the NPC and the potential effects of treatment on swallowing and speech, early involvement of nutritionists and speech therapists would be important.

The decision-making process of the MDT would also consider the patient's quality of life, potential treatment-related toxicity, and psychosocial aspects. The treatment plan would be tailored to the individual patient, taking into account his preferences and social support systems.

It's important to note that this response is based on the standard of care at the time of my last training data update in April 2023, and the latest clinical guidelines and research should always be consulted for the most current treatment recommendations.

**CASE 5: SALIVARY GLAND**

**Pretend you are the senior head and neck oncologist, chairing the multidisciplinary oncological group, which is deciding the best treatment for each patient with a new oncological diagnosis. Which treatment would you suggest for the patient thereafter described?**

**The patient is a 61-year-old man, with a recent diagnosis of poorly differentiated non-small cell epithelial neoplasia of the right parotid gland. Tumor staging is cT4a cN2b cM0. The patient has no history of smoking or alcohol consumption. The physical examination shows right parotid swelling of hard-woody consistency, fixed in relation to both superficial and deep planes, with involvement of the overlying skin without signs of fistulization. The patient has no other significant active disease and his medical record reports no previous surgeries, treatments, or significant conditions. The imaging performed for the tumor is as follows. The diagnostic ultrasound report is the following (in quotation marks):” Ultrasound reveals five hypoechoic nodular formations with irregular profiles and modest vascular signal in the right parotid gland (two in the superior position measuring 18.8 and 14.9 mm, and three contiguous to each other and adjacent to the former, located in the inferior position, measuring 9.5, 18, and 59 mm). Ipsilateral lymphadenopathies are present at levels II and III, the largest at level II with an oval hypoechoic appearance and a reactive-looking germinal center measuring 21 mm in its major axis. Hypoechoic fusiform and oval lymph nodes are noted at level III.” Contrast-enhanced magnetic resonance imaging report is the following (in quotation marks): “Multicystic lesion with a maximum diameter of 48 mm occupying a large portion of the right parotid gland, accompanied by lateral cervicallymphadenopathy at level II, the largest measuring 27 mm. A 12 mm lymph node is noted in the right supraclavicular fossa. The parotid lesion appears highly heterogeneous, even after contrast enhancement, with clear signs of diffusion restriction, indicative of a rapidly evolving condition.”**

**- Physical examination: Right parotid swelling of hard-woody consistency, fixed in relation to both superficial and deep planes, with involvement of the overlying skin without signs of fistulization.**

*This is a complex case of an advanced, poorly differentiated non-small cell epithelial neoplasia of the right parotid gland. Here's the breakdown of the situation and proposed treatment approach:*

*Understanding the Diagnosis*

- *Poorly-differentiated non-small cell epithelial neoplasia: This indicates a cancer originating from gland cells in the parotid gland. "Poorly differentiated" means the cancer cells are aggressive and likely to grow quickly.*
- *cT4a cN2b cM0: This is the clinical staging based on the size of the tumor and its spread:*
  - *T4a: Tumor has spread beyond the parotid gland, involving skin, bone, and other structures.*
  - *N2b: Cancer has spread to multiple lymph nodes on the same side of the neck.*
  - *M0: No evidence of distant spread of cancer (metastasis).*

*Treatment Approach*

*Due to the aggressiveness of this cancer and its advanced stage, a multimodal approach will be necessary:*

1. *Surgery: This is the primary treatment. The goal would be radical resection of the primary tumor with wide margins, along with a radical neck dissection (removal of lymph nodes in the neck). Reconstructive options may be needed.*
2. *Postoperative Radiation Therapy: Given the advanced stage and aggressive nature of the cancer, radiation therapy will almost certainly be necessary after surgery to decrease the risk of recurrence.*
3. *Chemotherapy: Depending on the exact pathology (a biopsy/surgical specimen analysis will clarify) and discussions within the multidisciplinary team, chemotherapy may be recommended either before surgery (neoadjuvant) to shrink the tumor or after surgery (adjuvant) to reduce the risk of recurrence further.*

*Important Considerations:*

- *Patient Counseling: The patient needs thorough discussion about the nature of his cancer, treatment options, risks and benefits, and potential side effects.*
- *Biopsy: A biopsy is needed to get a definitive diagnosis about the specific cancer type and help tailor treatment decisions.*
- *Expert Collaboration: A team including a head and neck surgeon, radiation oncologist, medical oncologist, pathologist, and other specialists is vital for optimal care.*

*Prognosis:*

*The prognosis for this patient is guarded given the advanced nature of the cancer. However, with a combination of aggressive treatment and close follow-up, many patients with advanced cancers have long-term survival.*

*Disclaimer: I'm an AI chatbot, not a medical professional. This information is not a substitute for a doctor's assessment and treatment recommendations.*

Given the detailed description of the patient's condition, the treatment approach for a patient with poorly differentiated non-small cell epithelial neoplasia of the right parotid gland, staged as cT4a cN2b cM0, would be comprehensive and multidisciplinary. This case presents a challenging scenario requiring a combination of surgical, medical, and possibly radiation therapy. Here’s an outline of a potential treatment strategy:

1. Surgical Intervention: The primary treatment for salivary gland cancers, especially those that are locally advanced and without distant metastasis (cM0), is surgical resection. Given the tumor's extension (cT4a), involving the parotid gland with skin involvement, and the presence of fixed lymphadenopathy (cN2b), a total parotidectomy with facial nerve preservation, if feasible, would be considered. Due to the skin involvement, resection of the overlying skin might also be necessary. The goal would be to achieve clear margins to reduce the risk of local recurrence. Neck dissection targeting the involved lymph node levels (II and possibly III, given the lymphadenopathy) would also be indicated to manage regional disease.
2. Radiation Therapy: Postoperative radiation therapy would likely be recommended due to the high-risk features of the tumor, including poor differentiation, advanced T stage, N stage involvement, and to address any microscopic disease that may not have been completely resected. Intensity-Modulated Radiation Therapy (IMRT) could be considered to maximize the dose to the tumor while minimizing exposure to surrounding critical structures.
3. Systemic Therapy: Given the aggressive nature of the tumor, adjuvant systemic therapy might be considered. This could include chemotherapy or targeted therapy, depending on the molecular characteristics of the tumor, if available. The role of systemic therapy in salivary gland cancers is evolving, and decisions would typically be based on clinical trial evidence or emerging treatment protocols.
4. Multidisciplinary Team Input: The management plan should be discussed in a multidisciplinary tumor board setting, including input from head and neck surgeons, medical oncologists, radiation oncologists, radiologists, and pathologists, to tailor the treatment plan to the patient's specific disease characteristics and overall health status.
5. Supportive Care and Rehabilitation: Given the potential impact on facial function, speech, and swallowing, the involvement of supportive care specialists such as physiotherapists, speech and language therapists, and dietitians would be crucial for postoperative rehabilitation and quality of life improvement.
6. Follow-Up and Surveillance: Regular follow-up with imaging and physical exams will be important to monitor for disease recurrence and manage any long-term side effects of treatment.

This proposed treatment plan is based on general guidelines and practices for managing advanced salivary gland cancers. Individual patient factors, tumor biology, and new emerging therapies should be considered in the final decision-making process.
